# Associations between Weight Loss, Food Likes, Dietary Behaviors, and Chemosensory Function in Bariatric Surgery: A Case-Control Analysis in Women

**DOI:** 10.3390/nu11040804

**Published:** 2019-04-09

**Authors:** Patrice A. Hubert, Pavlos Papasavas, Andrea Stone, Helen Swede, Tania B. Huedo-Medina, Darren Tishler, Valerie B. Duffy

**Affiliations:** 1 Department of Allied Health Sciences, University of Connecticut, Storrs, CT 06269, USA; patrice.hubert@uconn.edu (P.A.H.); tania.huedo-medina@uconn.edu (T.B.H.-M.); 2 Department of Surgery, Hartford Hospital, Hartford, CT 06102, USA; Pavlos.Papasavas@hhchealth.org (P.P.); Andrea.Stone@hhchealth.org (A.S.); Darren.Tishler@hhchealth.org (D.T.); 3 Community Medicine and Health Care, University of Connecticut Health Center, Farmington, CT 06030, USA; swede@uchc.edu

**Keywords:** taste, sweet liking, dietary behavior, gastric bypass, hunger, diet quality, preference

## Abstract

We tested the hypothesis that successful weight loss post-bariatric surgery would be associated with healthier chemosensory function, food likes, and dietary behaviors than either unsuccessful weight loss or pre-surgery morbid obesity. In a case-control design, pre-surgical women with morbid obesity (*n* = 49) were compared with those 1-year post-surgery (24 Roux-en-Y Bypass, 24 Sleeve Gastrectomy) and defined by excess or percent weight loss as successful/unsuccessful. For self-reported smell/taste perception, more post-surgery than pre-surgery reported improved/distorted perception, especially if weight loss successful. Measured taste function (perceived quinine and NaCl intensity) was lower among weight loss unsuccessful versus pre-surgery patients, yet a genetic variation in taste probe (propylthiouracil bitterness) matched expected frequencies without significant pre/post-surgery difference. Regarding survey-reported liking, higher diet quality was seen in the weight loss successful (independent of surgery type) versus pre-surgical patients, with differences driven by lower sweet and refined carbohydrate liking. The post versus pre-surgical patients had greater restraint but less hunger and disinhibition. Patients reporting both higher diet quality and lower hunger showed greater % weight loss, independent of surgery type. Thus, successful weight loss 1-year post-bariatric surgery was associated with improved or distorted chemosensation and patterns of liking associated with healthier diets, especially if coupled with less hunger.

## 1. Introduction

Obesity is a prevalent problem across the world. In many regions, women display a greater risk of severe obesity than males. For example, in the U.S. National Health and Nutrition Examination Survey (NHANES) 2015–2016, the prevalence of Grade 3 obesity (BMI ≥ 40.0) was almost double among women than men (10.0% versus 5.6%, respectively) [1]. Bariatric surgery serves as an effective way to achieve weight loss and improvements in metabolic health for those with severe obesity. Due to its low rate of complications, cost effectiveness, reduction of comorbidities, and demonstrated success in weight loss, the number of surgeries being performed has been on a steady rise [2,3]. In, 2017, there were 228,000 surgeries performed, a 5.6% increase from 2015 and 44.3% increase from 2011 [4]. In trend analysis from 2002 to 2011, women comprised nearly 81% of bariatric surgeries completed [5].

Bariatric surgery has been shown to reverse Type 2 Diabetes and decrease cardiovascular disease risk, including reductions in risk of hypertension and hyperlipidemia [3,6]. Of the surgeries performed, the most common are Roux-en-Y Gastric Bypass (RYGB) and Sleeve Gastrectomy (SG), at 17.8% and 59.4%, respectively [4]. The surgeries are estimated to result in an excess weight loss of 60–80% for RYGB and 50% for SG [2]. The level of weight loss from bariatric surgery is commonly reported as percent of excess weight loss (%EWL) and both surgeries have an estimated maintenance of 50%EWL [4]. However, there is no standardized definition of success from primary bariatric surgery [7] and %EWL has limitations [8,9]. One is that %EWL may not reflect successful weight loss in patients with very high BMIs due to the amount of weight discrepancy from ideal BMI. Patients with extreme obesity might show greater absolute weight loss, but still report a lower %EWL than patients with lower BMIs. A second limitation is that %EWL uses ideal body weight in the calculation, and due to inconsistent standards for calculation of ideal body weight, %EWL can vary [8,10]. The %EWL has ranged from 66% to 83% in the same population as a result of the various methods available to calculate ideal body weight [11]. Alternatively, percent weight loss (%WL) has been recommended as the new standard for reporting weight loss success. It has increased statistical advantages, is a more intuitive method, is the standard of reporting weight loss success in non-surgical weight loss studies for comparability, and is least associated with baseline BMI [10]. However, %WL has its limitations. For example, a patient with a higher weight would need to achieve greater weight loss than one who is of a lower weight to achieve the same clinical impact and approach normal weight range. The %WL clinically may not reflect weight loss success or failure [8]. Thus, examining both methods to calculate weight loss may improve the ability to examine factors associated with weight loss success from primary bariatric surgery.

Weight loss success in bariatric surgery can be attributed to their restrictive, and with the RYGB, malabsorptive nature [3] to suppress appetite and hunger, enhance satiety, change gut derived signals and sensory mechanisms to lower reward and preference for palatable foods [12], as well as alter the gut microbiota and bile acid [13]. The reward of food can be dissected into liking (hedonic impact), wanting, and learning [14], with liking as the primary driver of what individuals eat. Obese individuals may have a stronger hedonic response to foods than non-obese individuals [15,16], making weight loss challenging. Decreased liking or preference for energy-dense foods may result from changes in chemosensory function, gut-related hormones mediating food aversions [17], neural activation in reward-related brain areas, motivation to consume, and hedonic hunger [16,18,19,20].

Several review articles have highlighted human studies of changes in preference for energy-dense foods following bariatric surgery [19,21,22]. Some studies utilize reported food intake (e.g., dietary recall, food frequency surveys) to assess changes in food preference across bariatric surgery [19]. These measures are time-intensive for patients and clinicians, as well as frequently biased by misreporting [23]. Many studies have used survey-based assessment of food liking as a proxy for directly assessing preference with orally sampling single tastants (e.g., aqueous sucrose), simple food systems (e.g., milks with varying fat and sucrose added), or complex foods. For example, in a cross-sectional survey study with patients at different times pre- to post-surgery, the majority reported changes in preference, with recalled preferences for unhealthy foods that decreased and healthy foods that increased [24]. In response to sucrose solutions, pre- to post-bariatric surgery patients with approximately 20% body weight loss reported decreased preference for sweets, accompanied by improved ability to control intake of sweets [25,26]. In a progressive ratio task often used to test how hard laboratory animals will work for a reward, bariatric patients showed reduction in reward for a sampled sweet/fat mixture (candy), but no change in reward for a vegetable [27]. Direct preference testing with a buffet meal with self-selection revealed a decrease in total food consumed but no changes in food preference from 3 months pre-surgery to 6 months [28] and 18 months [29] post-bariatric surgery. The post-surgery patients who reported decreases in preference for energy-dense foods had the most success in total weight loss at 18 months [29]. Dietary guidance suggests that long-term healthy weight results from lower intake of high fat and sweet foods but enhanced consumption for healthier foods, such as whole grains, low-fat protein, fruits, and vegetables [30]. Consumption of a healthier diet, including reduction of unhealthy foods and increases in healthy foods, was associated with better weight loss and more favorable metabolic profile after bariatric surgery [31].

Direct measurement of preference using sampling of food is time and cost intensive, impractical for clinical care, as well as limited to the context of time and testing setting and the state of the participant in this setting. Asking the level of liking/disliking of sweet and salty shows reasonable association with orally sampled sweet and salty solutions, sweet or salty foods [32,33,34], as well as vegetables [35]. Survey-reported food liking also is associated with reported intake across a number of studies [36], as well as intake and craving for sweets [32]. Furthermore, survey-reported liking has been shown to correlate with nutritional biomarkers [37,38], supporting its association with habitual food intake. Liking surveys comprised of foods/beverages that reflect dietary guidelines can be formed into an index of diet quality similar to the Healthy Eating Index [39] with sufficient reliability, construct validity, and associations with risk factors of cardiovascular disease [38,40], including for individuals with morbid obesity [41].

The intersection between taste and smell (chemosensory) functions is complex. True taste (perception of sweet, salty, sour, bitter, umami) is confused with smell through the mouth (retronasal olfaction) in the processing of food flavor and the smells of foods (orthonasal olfaction). Functioning can be measured as sensitivity at the point of detection (e.g., detection threshold for sucrose) but sensitivity may have little relevance toward food preference, intake or behaviors (e.g., preference for sweet, intake of sweets, sweet food craving, respectively). Suprathreshold function captures what is perceived in the real world (e.g., perceived intensity of sweetness, ability to identify sweet from sour), yet the relationship with how much sweet is liked varies. For example, hedonic response to growing concentrations of sweet can classify individuals as sweet likers (increasing hedonic response) and dislikers (decreasing hedonic response) [42].

Most human studies do not support marked changes in measured taste function from pre- to post-surgery but do find that patients self-report changes in taste or smell function. Some studies find threshold improvement for sucrose [43], but there are not consistent pre-post-surgery changes across all basic tastes [25,26]. For suprathreshold, there were no significant changes in perceived intensity of sucrose or sodium chloride [26]. Most patients after bariatric surgery report alterations in taste perception, including repulsive and aversive responses, and that the taste alterations were associated with better weight loss [44,45,46]. However, the foods perceived as “tasting” different (distorted) or have aversive responses were complex chemosensory stimuli instead of basic tastes [45], demonstrating the common confusion between true taste and overall flavor sensation. Equal numbers reported improvements and reductions of sweet and salty tastes with bariatric surgery [45]. Changes in smell perception are less frequently reported [46], yet a pre-post bariatric surgery study found improvements in smell threshold and identification abilities [47].

What is unclear from the reported literature is if broader measures of suprathreshold taste function vary with bariatric surgery, and if there is an effect of taste genetics in the success from the surgery, including in reported food liking, dietary behaviors, and weight loss. Suprathreshold taste function measures were included in the 2011–2014 National Health and Nutrition Examination Survey [48]. The probes of quinine and NaCl intensities have been associated with adiposity in a large laboratory-based sample [49]. A commonly studied probe of taste genetics is the ability to taste the bitterness of propylthiouracil (PROP) [49] with reports on associations between PROP bitterness and food preference, dietary behaviors, and adiposity [50].

Long-term weight loss success also has been attributed to dietary behaviors characterized by higher cognitive control on eating, as well as less disinhibited and emotional eating [51,52,53,54]. Patients 1-year post-bariatric surgery report increased cognitive restraint of eating, decreased disinhibition, and reduced hunger [55,56]. Similar results were seen 4 months following RYGB [57]. Patients who exhibited a 1-year weight loss greater than 25% reported less hunger and disinhibition than those who did not achieve this level of weight loss [58]. There is potential synergy between changes in food hedonics and dietary behaviors with bariatric surgery. In a protein-modified fast intervention, greater weight loss success has been reported in patients with strong food-related inhibitory control, as well as low hedonic response towards food [51].

Thus, the primary purpose of this study was to compare chemosensory function, food liking, and dietary behaviors in women with morbid obesity prior to bariatric surgery and those at 1-year post bariatric surgery using a case-control design. We hypothesize that post-bariatric surgery patients will perceive improvements with the surgery, have greater chemosensory function, and report healthier dietary patterns and behaviors than the pre-bariatric patients. Secondly, we aim to examine differences in chemosensory function, food liking, and dietary behaviors between those who are more or less successful in weight loss at 1-year post-surgery and compared with the pre-bariatric surgery patients. We hypothesize that weight loss success will be associated with greater chemosensory function improvements, less liking for sweets and high-fat foods, as well as healthier patterns of food liking and dietary behaviors.

## 2. Materials and Methods

### 2.1. Participants

One-hundred females were recruited from patients in the Surgical Weight Loss Center at Hartford Hospital (A Bariatric Surgery Center of Excellence endorsed by the American College of Surgeons and the American Society for Metabolic and the Bariatric Surgery) to obtain 50 patients with morbid obesity who were pre-surgical (controls) and 50 who were 1-year post-bariatric surgery (cases), including 50 RYGB and 50 SG (planning in the pre-surgical group, underwent in the post-surgery group), stratified by the lowest and highest tertiles of excess weight loss. Only females were recruited because the majority of bariatric patients in this Center and nationwide [5] are female. Patients were offered bariatric surgery if they had a BMI equal or greater than 40 kg/m^2^ or a BMI between 35 and 40 kg/m^2^ with associated comorbid diseases. The decision between a RYGB or SG surgery was based on patient preference after a consultation with the surgical team, based on risk factors and medical profile. Exclusion criteria were current Axis I or Axis II mental health disorders, past medical history of Axis II disorder, Grave’s disease or thyroid problems, breastfeeding, and pregnancy. The study was approved by the IRBs at Hartford Hospital, UConn Health, University of Connecticut, and Connecticut Institute for Clinical and Translational Science. Approximately 350 patients were contacted and 100 met the criteria and stratification for participation. The pre-surgery patients were evaluated approximately 2 to 4 weeks pre-surgery. They were recruited at office visits by screening upcoming schedules and discussing the study on the phone, or by provider referral during their clinical appointment. To recruit the cases and to assure variability in 1- year post-surgical weight loss and equal numbers of RYGB and SG, 6-month post-surgery patients were identified from our IRB-approved database and characterized as high and low weight. Those with the highest (≥65%) and lowest tertile (<33%) excess weight loss were invited to participate at 1-year weight loss via mailings as a first attempt and subsequent call attempts (2 maximum, unless they indicated interest). Of the 100 patients total enrolled, 1 withdrew during taste testing and 1 patient died 6 months after surgery due to an acute MI. Participants provided informed and written consent and received a $20 Amazon gift card for participation.

### 2.2. Study Procedure and Measures

Participants completed all of the testing in a single session in a clinical visit. After the consent procedure, they completed the medical history form, self-reported chemosensory function survey, the liking survey, and the modified three-factor eating inventory. Next, trained technicians oriented them to the general Labeled Magnitude Scale [59], administered testing of a probe for retronasal olfaction and sweet preference, and conducted taste testing, including the ability to taste the bitterness of PROP.

Self-reported chemosensory function: The patients completed this paper or pencil survey that was based on the National Health and Nutrition Examination (NHANES) 2012–2014 chemosensory protocol [60], and modified for pre- and post-surgery reporting. The survey first asked about the sense of smell and then about the sense of taste, including: problems in the past year (y/n); describing their smell or taste; ability to smell, taste, or perceive food flavors now compared to when 25-years old; problems within the past year (check all that applied from problems perceiving, not perceiving things correctly, phantoms, stronger perceptions, smells make anxious or sick); when they noticed the problem; and suffering from chemosensory-related medical history (injury to head, neck, face; flu or head cold in past year; dry mouth; frequent nasal congestion). For post-surgery, the survey asked about problems, descriptions, and changes with the surgery.

Taste and retronasal testing: Participants rated the intensity of taste and flavor sensations on the general Labeled Magnitude Scale (gLMS), a 100-point scale with labels of intensity that are spaced in a logarithmic fashion and generalized to sensations of any kind [59], including 0 = ”nothing”, to 100 = ”strongest sensation of any kind,” and intermediate labels of 1.4 = ”barely detectable,” 6 = ”weak,” 17 = ”moderate,” 35 = ”strong,” and 53 = ”very strong.” Intensity ratings from the gLMS are consistent with those generated from magnitude matching [59]. Using a standardized procedure outlined in the NHANES protocol [48], participants were oriented to using the gLMS by rating the intensity of brightness of three remembered stimuli (for example, the brightness of a well-lit room). All of the participants were able to order correctly the intensity of a dimly-lit room < well-lit room < brightest light ever seen. The intensity of the well-lit room was used in the statistical analysis to partition out idiosyncratic scale usage.

Using a standardized procedure, participants rated the intensity of sweetness, flavor, and level of liking/disliking for cherry, coffee, and chocolate, as well as these sensations plus level of burning for Tabasco Gourmet^®^ jelly beans (Jelly Belly, Fairfield, CA, USA). The participant was told to plug her nose and rate the intensity of sweetness (and burn for Tabasco) only, and then unplug her nose and rate the intensity of sweetness, burn (Tabasco only), flavor (label provided), and level of liking/ disliking. The intensity of sensations and level of liking/disliking was compared between the groups and the jelly bean liking was compared with the survey-reported liking of sweets.

Taste function and PROP tasting: Regional and whole mouth taste function were assessed in a procedure validated in the NIH Toolbox for Assessment of Neurological and Behavioral Function project [61] and further standardized in the NHANES protocol [48]. First, participants provided intensity ratings of aqueous solutions drawn across the tongue tip, testing taste and oral irritation from the chorda tympani branch of cranial nerve VII for concentrated 1 mM quinine hydrochloride (QHCl) and 1 M sodium chloride (NaCl). Participants then rated the intensity of whole mouth 1 mM QHCl and 1 M NaCl. Finally, as a probe of genetic variation in taste [62,63], they rated the intensity of 1 mM and 3.2 mM PROP sampled with the whole mouth, with the rating of 0.32 M NaCl in between the two PROP solutions. Participants rinsed before and after each tastant with tap water and expectorated all rinses and tastants after sampling. The taste intensities were treated as continuous for comparison between pre- and post-surgery and between successful and unsuccessful groups. Additionally, PROP intensity relative to NaCl intensity [62] was used to define non-tasters, medium tasters, and supertasters for comparison to expected frequencies of 25%, 50%, and 25%, respectively, and to compare between the groups. However, because of the abbreviated protocol, we used only a ratio of 1 mM PROP to 0.32 M NaCl in addition to the intensity of 3.2 mM probe. For classification as a nontaster, participants reported the 3.2 mM PROP ≤20 (around moderate), the 1 mM PROP <17 (less than moderate), or had a ratio of 1mM PROP to 0.32 NaCl <0.4. For classification as a supertaster, participants reported 3.2 mM PROP ≥55 (greater than very strong), 1 mM PROP >35 (greater than strong), a ratio of 1mM PROP to 0.32 NaCl at ≥1, and reported an increase in perceived intensity from 1 mM to 3.2 mM PROP. The medium taster classification applied to those who rated the 3.2 mM PROP as between 20 and 55 and did not meet the criteria for either nontasters or supertasters. 

Liking Survey: Participants completed a validated, 100-item liking survey comprised of foods, beverages, physical activities, sedentary activities, pleasant experiences, and unpleasant experiences [41]. The scale for items ranged from −100 to 100, indicating “strongest disliking of any kind” (-100) to “neutral” (0), to “strongest liking of any kind” (+100). Each item displayed a word label and pictures corresponding to the word, and participants were asked to mark on the scale their level of liking. The scores were then measured using a ruler to indicate the “amount” of liking by the participant. The items were averaged and formed into eighteen groups, indicating liking for that group: nutritional groups (alcohol, sweet foods, fruits, vegetables, low-fat protein, high-fat protein, sweet drinks, fats, refined carbohydrates, fiber-rich, salty), sensory groups (bitter, sour, spicy/ flavorful), physical activities, pleasant experiences, and unpleasant experiences. These groups were used to form average scores for healthy (fruit, vegetable, low-fat protein, and fiber-rich foods) and unhealthy (sweet foods, high-fat protein, sweet drinks, fats, refined carbohydrates, salty) liking. Finally, a diet quality index (DQI) was calculated from 10 of these food groups with a positive weight for healthy groups and negative weight for unhealthy groups following the 2015 Dietary Guidelines: sweet drinks (−3), sweet foods (−3), fruits (+2), vegetables (+3), refined carbohydrates (−1), fats (−2), low-fat proteins (+3), high-fat proteins (−3), salty foods (−2), and fiber (+2). A higher score on the liking scale corresponded with a higher diet quality score and being healthier. A Healthy Behavior Index (HBI) was also created by adding physical activity to the diet quality index.

Dietary Behavior: A modification of the Three Factor Eating Questionnaire (TFEQ) [64] was used to assess cognitive restraint of eating, disinhibition, and hunger. The original survey derived these constructs from factor analysis of data from several populations with various levels of dietary restraint. We used the first 36 items from the original questionnaire and 13 were adopted from two validated subscales of the questionnaire, which measured flexible and rigid control [65]. These items were reported as “True” or “False”, except for the last 2 items on eating until full, with 3 choices (I quit before I feel full, I quit when I feel full, I continue despite feeling full), and how fast you normally eat, with 5 choices (very slow, relatively slow, medium, relatively fast, very fast). Items were scored according to the original criteria [64,65] for each participant for dietary restraint, disinhibition, and perceived hunger.

Surgical Procedures and Calculating Weight Loss: All procedures were performed by two bariatric surgeons (P.P., D.T.) using the same laparoscopic technique for SG (34F gastric lavage tube) and different techniques for RYGB (laparoscopic retrocolic, retrogastric versus robot-assisted antecolic, antegastric). The study site is accredited by the Metabolic and Bariatric Surgery Accreditation and Quality Improvement Program (MBSAQIP). All patients were instructed to follow the same post-operative low carbohydrate, low fat diet.

Two methods were used to characterize successful versus unsuccessful weight loss post-bariatric surgery. Percent excess weight loss (%EWL) was calculated using the standardized method, which takes into account the ideal weight for the patient: ((Preop Weight) − (1 Year Weight))/(Preop Weight) − (Ideal Weight)) * 100 [8]. Criteria for successful weight loss by %EWL method varies greatly [7], including weight loss > 50% [66] to 60% [67]. The present study used the highest tertile of weight loss from an IRB-approved database, which was 65%EWL, as the criterion to define successful versus unsuccessful. Percent weight loss (%WL) was calculated as: ((Preop Weight-1 Year Weight)/Preop weight * 100). The criterion for successful weight loss by %WL is considered > 27% [68], and was used in the present analysis as the criterion for successful versus unsuccessful.

### 2.3. Statistical Analysis

Data were analyzed using SPSS (version 24, IBM Corporation, Chicago, IL, USA) and R Software (3.4.1 with Lavaan, University of California, Los Angeles, CA); significance level was set at *p*-value ≤ 0.05. Differences between surgery groups were assessed based on t-tests or chi-square analyses. Cronbach’s alpha was used to test the internal reliability of composite variables. Associations between variables or between covariates were assessed by the Spearman rho statistic or standard linear regression analysis, based on the normality of the distribution and data transformation if required. Descriptive statistics and chi-square analyses were used to evaluate the self-reported chemosensory function, comparing pre-surgical patients with the NHANES 2012/2014, describing changes in chemosensory function in the post-surgical women, and changes between successful and less successful with weight loss post-bariatric surgery. Inferential statistics examined the differences between the pre- and post-surgical women, and pre-surgery and success post-surgery. Analysis of covariance (ANCOVA) was used to assess difference in chemosensory function, food liking, and dietary behaviors between the cases (post-bariatric surgery) and the controls (awaiting bariatric surgery for morbid obesity), as well as between those successful and less successful with weight loss versus the pre-operative surgical group, controlling for surgery type, pre-operative weight, age, remembered sensation (for analyses with the intensity measures), or non-food liking (for analysis with the liking survey) and PROP tasting (for analyses with the taste measures) [63]. The assumptions of ANCOVA were met, including assuring that the covariates did not show strong correlation, evaluation of the normality or outliers at each level of the independent variable (pre-surgery, post-surgery), visual inspection of the linearity between the covariates and the dependent variable for each level of the independent variable, and the Levene’s test for the equality of variances across the levels of the independent variable. If the Levene’s test did not support equality of variances, the Brown–Forsythe test was reported with Games-Howell post-hoc testing.

### 2.4. Power Analysis

Difference in chemosensory function, food liking, and dietary behaviors were the primary outcomes. Based upon variation in outcome related to taste status in our prior studies [38] and others [24], we had 80% power to detect a medium effect size (Cohen’s d = 0.3) at the 0.05 significance level, with three independent groups numbering between 20 and 25 participants per group.

## 3. Results

### 3.1. Demographics of the Participants

Table 1 displays the participant characteristics for pre- and post-surgery, as well as the post-surgery group categorized by either %EWL or %WL. The pre- and post-surgery groups did not differ significantly on age, race, ethnicity, or pre-operative adiposity. Following standardized reporting of weight loss at 1-year post-surgery [8], change in BMI averaged 12.9 (range 2 to 30.0), %WL averaged 27.2% (range 0 to 49.6%), %EWL averaged 65.6%, ranging from 12.0 to 140.3%.

Table 2 shows the agreement and disagreement between classifying weight loss success by these two methods. Of the 48 post-surgical patients, 11 were classified differently between the two methods. The %EWL groups differed significantly in baseline weight; as expected, both post-surgical groups differed in post-surgery weight.

### 3.2. Self-Reported Chemosensory Function

For self-reported smell function (Table 3), the percent of females with morbid obesity (pre-op) compared with the nationally-representative NHANES dataset (ages 40+ years) was similar for problems in the past year but different for changes since 25 years of age (χ^2^(2) = 12.72; *p* < 0.01). More females with morbid obesity reported a better sense of smell and fewer reported worse; the specific problems included distorted (things don’t smell right) or phantom smells, or that smells make them sick or anxious. In the post-surgery group, most reported no problems with the sense of smell since the surgery in a dichotomous question. However, with regard to a specific problem, more of the unsuccessful patients reported less ability to smell since the surgery and more of the successful patients reported distorted or phantoms smells. This was significantly different for the post-surgery patients defined by %WL and compressing the parosmia/phantom and stronger/sick anxious smell sensations (Fisher exact, *p* < 0.01).

For self-reported taste function (Table 4), the percent of females with morbid obesity (pre-op) compared with the nationally-representative NHANES dataset (ages 40+ years) was similar for problems in the past year, but more reported a better ability to taste across all taste qualities since 25 years of age (chi squares, *p* < 0.05). The females with morbid obesity were matched with the NHANES sample for ability to taste food flavors since a younger age. In the post-surgery group, the majority of patients reported no taste problems, but more patients reported taste than smell problems since the surgery (26 to 28% versus 5 to 11%, respectively, in Table 4 compared with Table 3). A significantly greater percentage of the successful group reported better ability to taste across the taste qualities, whereas a greater percentage of the unsuccessful group reported worse ability (chi squares, *p* < 0.01). In addition, patients in the successful group were significantly more likely to report that things did not taste right (distorted) and in the less successful group that they could not taste some things. This difference was significant for the group defined by %EWL (Fisher exact, *p* < 0.01). Only 1 patient in the post-surgery group reported tasting things when nothing should be there (i.e., phantom sensation).

The pre-operative and post-surgical patients, for either group, did not differ significantly in the sum of the number of chemosensory-related medical history (injury to head, neck, face; flu or head cold in past year; dry mouth; frequent nasal congestion) (*F*(1, 96) = 1.60, *p* = 0.21).

### 3.3. Sweet Taste, Retronasal Probe, and Sweet Liking

The perceived sweetness of the jelly beans ranged from moderate (Tabasco^®^ flavored) to just below strong (cherry flavored) and formed a reliable group (α = 0.71). In analysis of covariance (ANCOVA), controlling for covariates, the average perceived sweetness did not vary significantly across the pre- and post-operative groups or between either successful/unsuccessful weight loss groups (*p*’s 0.2 to 0.4). The intensity of burn from the Tabasco^®^ jelly beans averaged very strong, and also did not vary significantly across the pre-operative and successful/unsuccessful post-operative grouping in ANCOVA (*p* values 0.6 to 0.7). Likewise, the retronasal flavor of the jelly beans ranged from above moderate (chocolate) to above very strong (Tabasco^®^) and formed a reliable group (α = 0.77); the average flavor intensity did not vary significantly across the pre-operative or either successful/unsuccessful grouping in ANCOVA, controlling for covariates (*p* values 0.8 to 0.9). Likewise, reported liking for the jelly beans did not vary significantly across pre-operative and post-surgery groups (F(1,96) = 1.51, *p* = 0.22)), but was lowest in those successful with weight loss and significantly lower than that of the pre-operative group, whether classified by %EWL or %WL. For example, there was an overall effect trend for the %EWL (F(2,96) = 2.47, *p* < 0.1)), but significant pairwise comparison between pre-surgery and successful weight loss (−8.40 ± 6.3 versus 7.12 ± 4.1, *p* < 0.05). There was less overall effect for the %WL groups (F(2,96) = 1.44, *p* = 0.24)) and only a trend for pairwise comparisons (*p* = 0.1).

### 3.4. Taste Function and PROP Tasting

According to PROP taster classification, the pre- and post-surgery groups did not differ significantly in the percentages of non, medium, and supertasters (χ^2^ = 2.53, *p* = 0.28). Overall, the pre- and post-surgery groups were relatively close to the theoretical frequencies of 25% nontasters, 50% medium tasters, and 25% supertasters. The frequencies of taster groups did not vary significantly between pre- and post-surgery groups or between successful and unsuccessful weight loss groups (Table 5). Neither the intensity of 3.2 mM or 1 mM PROP varied significantly across surgery or weight loss groups (*p* > 0.25).

**Figure 1:**
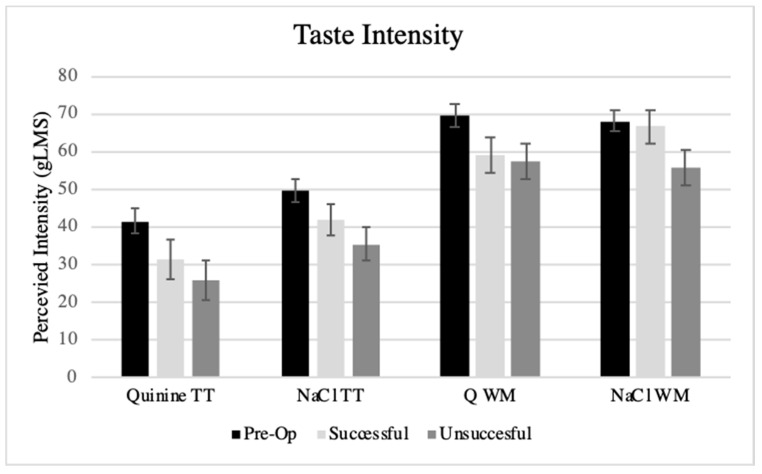
Perceived intensity of 1mM quinine tasted on the tongue tip (TT) or whole mouth (WM), as well as 1 M sodium chloride (NaCl) in women with morbid obesity waiting for bariatric surgery, and women 1-year post-surgery classified as successful versus unsuccessful in weight loss based on percent excess weight loss (gLMS 0 = ”no sensation”, 6 = ”barely detectable”, 17 = ”moderate”, 35 = ”very strong”, 54 = ”strongest sensation of any kind”). Across all tastants, there was significant differences between the pre-operative group and the unsuccessful group (*p* < 0.01).

Across all participants, the intensity of 1mM quinine on the tongue tip averaged strong (35.1 ± 2.65), and 1M NaCl averaged between strong and very strong (44.0 ± 2.25). Comparatively, water averaged near barely detectable (2.31 ± 0.56). Upon visual inspection, patients who reported intensities to water on the tongue tip above “weak” did not report having a dysgeusia or that things did not taste right. Whole mouth NaCl averaged between strong and very strong (44.03 ± 2.25) and quinine averaged above very strong (63.80 ± 2.46). Independent of the perceived intensity of remembered sensations, the perceived intensity of these tastants showed significant correlation with the bitterness of PROP. The r values ranged from 0.23, *p* < 0.05 for quinine on the tongue tip to 0.45, *p* < 0.01 for whole mouth quinine. Figure 1 shows the average intensities of tastant and location by surgery group defined by %EWL. In a repeated measure ANCOVA, controlling for covariates, there was a significant effect between pre- and post-surgical groups (F(1,90) = 5.86, *p* < 0.05)), with the post-surgery group reporting lower intensities across all tastants. Similar findings were seen in ANCOVA, with a taste factor averaged across the two tastants at tongue tip and whole mouth, explaining 70% of the variance across all of the participants and good internal reliability (α = 0.83). Further examination by groups defined by %EWL also showed significant effects on taste intensity ratings (F(2,89) = 3.86, *p* < 0.05), with significant differences between the pre-operative group and the unsuccessful group (*p* < 0.01). The unsuccessful group reported lower intensities than did the pre-operative group. Similar results were identified, whether grouping by %EWL or %WL. The total number of chemosensory risk factors did not explain significant variation in taste intensity.

### 3.5. Liking Survey

Across all patients, the groups (Table 6) ranged in liking from above strongly (fruit, pleasant, low-fat protein, high-fat protein groups), strongly to moderately (fat, fiber-rich, vegetable, sweets, refined carbohydrate, salty), moderately to weakly (physical activity, sour, bitter), and barely liked and disliked (sweet drinks, spicy/flavorful, alcohol, unpleasant). The liking-based groups had internal reliability that ranged from good to acceptable for 8 of 17 questions, questionable for 3 of 17, poor for 4 of 17, and unacceptable for 2 of 17 groups. The variance within the groups was highest for the alcohol, sour, sweet drinks, sweets, physical activity, bitter, spicy/flavorful, and low-fat protein groups. There was consistency between the rated liking of sampled jelly beans and survey reporting. The association between the average liking of the jelly beans (cherry, chocolate, coffee) and survey-reported liking for sweet drinks and sweet foods was significant (β = 0.36, *p* = 0.001 and β = 0.35, *p* < 0.01, respectively), as was the association between liking of the Tabasco^®^ jelly bean and the spicy or adventurous group (β = 0.41, *p* < 0.001).

**Figure 2:**
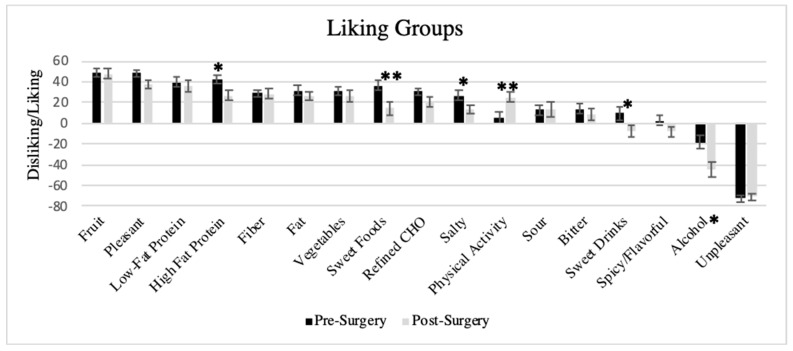
Average liking ± SEM for nutritional, sensory, and non-foods among women with morbid obesity awaiting bariatric surgery (pre-surgery) versus women 1-year post-bariatric surgery, listed from most to least liked; * *p* ≤ 0.05; ** *p* < 0.01.

Figure 2 displays the average liking of sensory, nutritional, and non-food groups between pre-and post-surgery patients. The order from highest to lowest liked group is very similar to what we found in a separate group of men and women with morbid obesity considering bariatric surgery [41]. Nearly 60% of women reported that fruit was more liked that pleasurable non-foods. This percentage did not vary significantly between those pre- and post-surgery. In t-tests, pre- and post-surgery groups showed significant differences in average liking for sweet, high-fat protein, salty, alcoholic beverage, and physical activity groups.

The food groups (except for alcoholic beverages) were constructed into a dietary quality index (DQI). Exploratory factor analysis (EFA) of these food groups showed that they produced two factors that could be conceptually labeled as less healthy and healthy, which together explained nearly 50% of the variance across the pre- and post-surgical patients. In separate EFA, adding the physical activity group increased the total variance explained to 57%. The internal reliability of the DQI constructed from these foods groups was good (α = 0.81) and showed normal distribution. Adding in the physical activity to the DQI (Healthy Behavior Index, HBI) produced similar reliability and normal distribution (not shown). The EFA results and distribution of the indexes are consistent with our previous findings with a separate group of women with morbid obesity [41]. 

**Figure 3:**
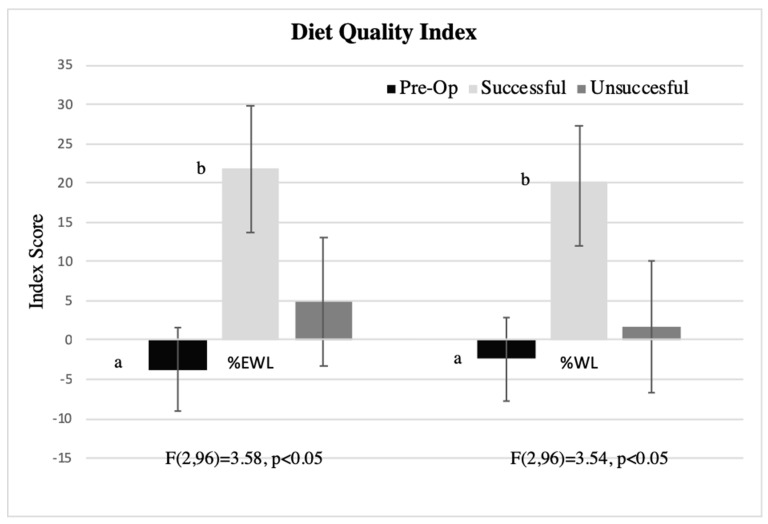
Diet quality index (DQI) scores based on food liking/disliking ratings in women with morbid obesity waiting for bariatric surgery (pre-op) and 1-year post-surgery defined as successful or unsuccessful based on % excess weight loss (left) and % weight loss (right). Unlike letter notations, a and b = significant difference, all *p* < 0.005).

Using ANCOVA, controlling for covariates, post-surgery women had significantly healthier DQI (F(1,96) = 5.13, *p* < 0.05) and HBI (F(1,96) = 8.50, *p* < 0.01) indices. Sub-analysis shows that these differences were driven by significant difference in liking of unhealthy foods (F(1,95) = 6.35, *p* = 0.01)). Liking for healthy foods did not show a mean difference between pre- and post-surgery groups (*p* = 0.72). Comparison of pre-operative with post-surgery categorized by success in weight loss group reveals significantly healthier DQI in the successful post-surgery versus the pre-operative groups (Figure 3). The unsuccessful group was not significantly different from either group. Similar findings were seen with the HBI—the successful group had significantly healthier indexes than the pre-operative group, defined by %EWL (F(2,96) = 5.51, *p* < 0.01 with pairwise comparison *p* < 0.005)) or %WL (F(2,96) = 5.60, *p* < 0.01 with pairwise comparison *p* < 0.005)). The effect of surgery type (SG or RYGB) was non-significant in all of these models (*p* > 0.6).

### 3.6. Dietary Behaviors

The EFA revealed three factors similar to the TFEQ constructs of dietary restraint, disinhibition, and hunger [64]. Similar to another study of individuals with morbid obesity [69], these factors only explained 34% of total variance. Based on the factor loadings, some questions moved from one factor to another or were not included. The analyzed factors had fair to good internal consistency for dietary restraint (17 questions, α = 0.756), disinhibition (19 questions, α = 0.890), and hunger (10 questions, α = 0.838).

**Figure 4:**
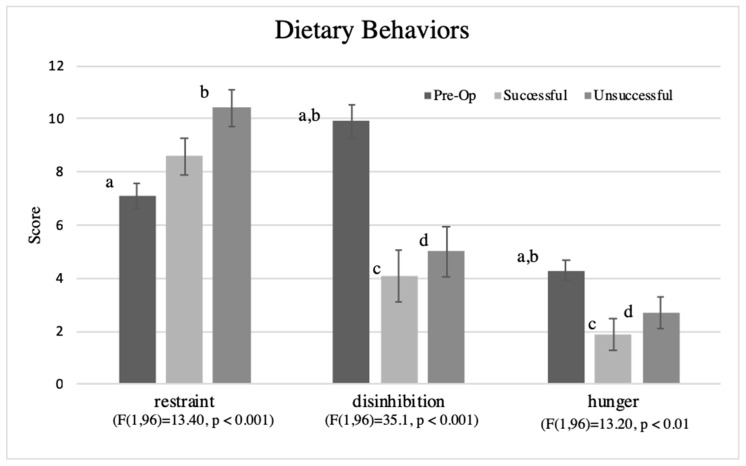
Average scores on restraint disinhibition, and hunger among women with morbid obesity before bariatric surgery (pre-op) and those 1-year post-surgery, defined by percent excess weight loss into successful (≥65%) and unsuccessful (<33%) weight loss groups. Unlike letter notations within a variable (a versus b; a,b versus c,d) = significant difference, all *p’s* < 0.05).

In comparing the surgery groups in ANCOVA controlling for covariates, the pre-op group had significantly lower restraint (F(1,96) = 13.40, *p* < 0.001), higher disinhibition (Brown-Forsythe (F(1,96) = 35.1, *p* < 0.001), and higher hunger (Brown-Forsythe (F(1,96) = 13.20, *p* < 0.01) than the post-surgery group (Figure 4). Sub-analysis by surgery weight loss success showed that dietary restraint was significantly higher among unsuccessful post-surgery than pre-operative group, disinhibition was significantly lower among successful and unsuccessful weight loss groups than pre-operative group, and hunger was significantly lower in both surgery groups than the pre-operative group. These results are shown for the groups defined by %EWL (Figure 4), which are similar for those defined by %WL.

The dietary behaviors showed significant association with DQI and HBI, including higher DQI/HBI and higher restraint (rho = 0.24/0.29, *p* < 0.05), lower DQI/HBI, and higher disinhibition (rho = −0.51/−0.54, *p* < 0.01), lower DQI/HBI and higher hunger (rho = −0.36/−0.37, *p* < 0.01).

### 3.7. Associations between Liking-Based Indexes, Dietary Behaviors, and Percent Weight Loss in Post-Surgical Patients

In separate multiple regression analyses, controlling for covariates, a greater level of weight loss was associated with either a higher diet quality (β = 0.21, *p* < 0.05), healthy behavior index (β = 0.21, *p* < 0.05), and less hunger (β = −0.21, *p* < 0.05), but not with more restraint (β = 0.12, *p* < 0.2) or less disinhibition (β = −0.12, *p* = 0.22). Due to the correlation between the liking-based indexes and hunger, adding both in the multiple regression analyses suppressed either effect. Instead, the joint influence of liking-based indexes and hunger was examined by creating concordant and discordant groups split at the median for each variable. There were 15 women who had low DQI/low hunger and 26 who had high DQI/high hunger, with 33 who had low DQI/high hunger, and high DQI/low hunger. In ANCOVA controlling for covariates, there was an overall trend for the concordant and discordance groups (F (1,84) = 2.65, *p* = 0.06)). The greatest weight loss was observed in the high DQI/low hunger group and significantly greater than either the low DQI/low hunger (*p* < 0.05) or low DQI/high hunger groups (*p* < 0.05).

## 4. Discussion

Our case-control investigation describes chemosensation, food liking, and dietary behaviors in women with morbid obesity scheduled for bariatric surgery and women 1-year post-bariatric surgery (Sleeve Gastrectomy or Roux-en-Y Bypass), who were successful or unsuccessful at weight loss. The study is unique, as it included valid and standardized assessment tools of chemosensory function, an evaluation of genetic variation in taste, and valid dietary measures for individuals with morbid obesity. For example, the measure used to assess self-reported chemosensation was content validated and underwent cognitive testing for full implementation in the NHANES 2011–2014 [48]. The taste measure was validated and standardized in the NIH Toolbox project [61] and NHANES [48] as a brief, yet valid measure of taste function with relevance to nutrition and health outcomes. Furthermore, patients were assessed for the ability to taste the bitterness of propylthiouracil (PROP), the most common probe of genetic variation in taste that has been shown to associate with food liking, dietary behaviors, and adiposity [50,70]. We were unable to identify any published literature on PROP tasting and response to bariatric surgery, either dietary behaviors, or weight loss. 

Although most of the post-surgery patients did not report a smell or taste problem, those more successful with weight loss reported a greater frequency of distorted or phantom smells and better or distorted taste perception. In measured functioning, the women unsuccessful with weight loss perceived lower intensity to concentrated bitter and salty solutions than the pre-operative group, but did not differ in sweetness or olfactory flavor (retronasal olfaction) from a candy probe. We did not find that women with morbid obesity were more likely to be nontasters or supertasters of PROP compared with population estimates, or that the frequency of these taster groups varied significantly across the pre- or post-surgery groups. The weight loss successful group also reported a healthier pattern of food liking, including liking of physical activities. Although the post-surgical group reported significantly more dietary restraint, but less disinhibition and hunger, these dietary behaviors alone did not characterize women more successful in bariatric surgery weight loss. However, lower perceived hunger coupled with a healthier pattern of food liking was associated with a greater level of weight loss. The chemosensory, food liking, and dietary behavior findings in the present study were seen in the pre- versus post-surgical groups, whether defining weight loss success as percent excess weight loss or percent weight loss with the bariatric surgery.

The present study found less self-reported changes in taste and smell 1-year after bariatric surgery than that reported in other studies [44,45,46]. These changes may be initially noted after the surgery but become less so as time proceeds [24], or improve from immediate pre-surgery to 6-months post-surgery [47]. We did join other studies in reporting greater success with weight loss amount in patients who report altered taste and olfactory sensations [44,45,46]. We identified 2 to 7 times the rates of distorted or phantom smells or tastes in the successful versus unsuccessful weight loss groups. The reason for the distorted (parosmia) or phantom smell is unknown, but could suggest olfactory improvements with weight loss as reviewed [71], but that the improvements are not complete and the odor quality is distorted or not perceived as usual. Reporting that things do not taste right could actually be a confusion between taste and retronasal olfaction. Thus, the patient may perceive basic tastes correctly, but the composite flavor sensation of common foods is not correct, as previously noted [24]. Individuals with oral sensory nerve damage also can report altered taste and flavor sensations or phantom sensations [72], or a form of dysgeusia (persistent salt, sweet, sour, or bitter taste in the mouth). However, the pattern of taste perception from the tongue tip (chorda tympani branch of cranial nerve VII) in the present post-bariatric surgery patients (Figure 1) does not suggest damage to taste as the basis for these altered sensations. Dysgeusia also can be a persistent taste that is actually tasted, such as that caused by reflux. Gastroesophageal reflux, a side effect of morbid obesity as well as bariatric surgery [73], especially the sleeve gastrectomy [74], could contribute to a perception that things didn’t taste right or dysgeusia. The present study also was consistent with a previous report [45] that showed that post-bariatric surgery patients, especially if successful in weight loss, reported that they were better able to detect basic taste qualities. The taste and sensory quality of food can be perceived as more intense with slower rates of eating after bariatric surgery. Slower eating allows sufficient chewing and pressure to release and pump flavor volatiles from foods retronasally to olfactory receptors for full flavor perception [75]. Taste and retronasal olfaction contribute to the perception of more intense flavor sensations [72]. Rapid eating with less chewing after bariatric surgery would not improve flavor sensation and may be a sign of inappropriate dietary behaviors, emotional eating, and risk of weight regain [76].

Our measures of chemosensation did not support that women post-bariatric surgery have better taste or retronasal ability than those who have morbid obesity. The present study findings are consistent with other studies that employed similar psychophysical measures in a prospective data collection from pre- to post-bariatric surgery [25,26]. While there may be some improvements in taste threshold [43], this shift may not have relevance to the perception of real-world stimuli, and thus dietary behaviors. We did find that those less successful in weight loss reported lower taste intensity across concentrated bitter and NaCl stimuli. Measures of overall taste functioning may explain differences in food liking, intake, and risk of diet-related diseases, as shown by our laboratory [38,49,77,78] and others [79]. The present study design cannot determine if the lower taste function among the less successful weight loss group was just chance or related to the surgery. We also did not find that women with morbid obesity were more likely to be nontasters of PROP, or that the distribution of nontasters to supertasters varied significantly from expected frequencies and by surgery group. Nontasters of PROP report less intense sensations from foods and beverages, greater preference for, and intake of, sweets, high-fat, and alcohol, as well as greater risk of obesity [50,70]. There have been ties between the non-taster recessive haplotypes of TAS2R38, the taste receptor gene that mediates the ability to taste PROP, and greater risk of obesity [80]. Although we did see the expected relationship between PROP bitterness and the intensity of quinine and salt as reported [15], morbid obesity challenges that ability to measure the phenotype and see the PROP nontaster phenotype-obesity relationship.

The present study found more healthy patterns of food liking among those women who were more successful at bariatric surgery. This agrees with previous studies that report less liking for sweets and high fat food across bariatric surgery [24,25,26,37,81,82], including the association with greater success in weight loss [29] and more favorable metabolic profile [31]. We showed reduction of liking of sweetness in the candy probe and with survey liking of sweets and sweet drinks (i.e., unhealthy foods), as well as significant correlations between the liking of sample candy and survey liking of sweet (sweet candy) and spicy or flavorful (spicy candy) foods. The liking survey is feasible for a clinical setting, taking minutes to complete, and if online, limited time to process. Our work extends previous findings [24,25,26,37,81,82] through use of this feasible clinical measure that can provide a valid and reliable index of diet quality and health behavior. Our liking survey was formed into a diet quality index with construct and criterion validity similar to the Healthy Eating Index [39], and replicated our work with a separate sample of women with morbid obesity in a bariatric treatment setting [41]. Even though direct measurement of preference is supported to be more precise [21], we found a bigger effect between surgery groups with survey-reported food liking than preference for the sampled candy. Our liking survey contained non-food items, which following the principal of sensory standards [15], generalizes the scale for reporting the level of liking/disliking beyond foods, improving the ability to make comparisons across individuals. The association between the liking survey groups and overall diet quality index and biomarkers of nutritional status [37,38,40] supports its use as a proxy for habitual dietary intake, as well as measure of food liking. Change in diet quality in our sample was driven by the decreased liking of unhealthy foods, rather than an increase in liking of healthy foods. Similar results have also been seen [31], where decreased consumption of unhealthy food following RYGB improved dietary patterns.

Our study found improved dietary behaviors from pre- to post-bariatric surgery, but only perceived hunger associated with weight loss success, especially in concert with liking a healthier dietary pattern. Our findings are consistent with previous reports of increased cognitive restraint of eating, decreased disinhibition, and reduced degree of hunger [55,56,57,58,83]. Our study, as well as others [69,84], noted that the factor structure of the dietary behaviors generated from The Three Factor Eating Questionnaire [64] were not generalizable to women with morbid obesity or those who underwent bariatric surgery. Other studies have not found that post-surgery dietary behaviors, including restraint, were associated with the level of weight loss [58,85]. We were surprised to see that unsuccessful patients reported higher levels of restraint than pre-operative patients, as restraint is purported to be an indicator of weight loss success. Cognitive restraint may be more difficult to measure due to the surgery changes that make dietary restriction and avoidance of maldigestion and intolerance necessary [58]. It may be that unsuccessful patients require higher restraint because they are trying to lose weight, especially in the case of the present study, as they did not report healthy patterns of food liking that were different from the pre-operative group. Bariatric surgery patients are known to have a history of chronic dieting [86], which may have transformed into high levels of restraint in our study sample regardless of surgery success. Research has also suggested that high restraint may complicate weight loss [87,88]. The restraint theory suggests that those who restrain their eating are more likely to overeat, especially when their self-control is impacted by outside events, leading to disinhibited eating [89]. However, recent neuro-imaging research supports that bariatric surgery (RYGB)-related decreases in liking for unhealthy food and increases in liking for healthy foods results from changes in the frontoparietal control network, which involves cognitive control of food sensations, and failed to find involvement of reward-related brain regions [82]. Thus, it may be important to examine both changes in food liking as well as improved dietary measures in weight loss success from bariatric surgery.

The present study had a number of limitations and strengths that need consideration. The major limitation was the cross-sectional analysis, which prohibits cause and effect conclusions. The study findings may not apply beyond females. The self-reported chemosensory function and taste tests were standardized and consistent with methodologies in the NHANES 2011-2014 wave [60]. However, we only included a simple probe of retronasal olfaction and not full olfactory testing. The study included both direct and survey measures of food liking, although the direct measure of food liking was very brief and not as sophisticated as a complete meal testing [28,29]. Despite completing confirmatory and exploratory factor analysis, the dietary behavior factors did not explain a majority of the variance across the survey questions, a finding also reported previously [69]. This suggests the need to research dietary behavior constructs among individuals with morbid obesity who undergo obesity treatments, as well as using direct measures of inhibitory control [51]. Finally, we included patients only 1-year after bariatric surgery, whereas a longer follow-up is suggested to better understand characteristics of weight loss success and the food preference and dietary behaviors needed to maintain a healthy weight and metabolic profile. However, as a strength, success following surgery was identified as 1-year bariatric surgery weight loss at > 65%EWL, which is a higher success criterion than other studies that define success as > 60% [67] or > 50% [9]. The present study also used %WL as an outcome and the criterion of successful 1-year weight loss of > 25%, as recommended for high sensitivity and specificity against the gold standard of weight loss success [90].

## 5. Conclusions

This observational study supports that patients who are successful in weight loss 1-year post-bariatric surgery report improvements or alterations in their taste and flavor perception, as well as less liking of sweets and unhealthy foods and a pattern of liking reflective of a healthy diet. Liking of a healthy dietary pattern is coupled with less perceived hunger and associated with higher percentage weight loss. These findings support that the bariatric surgery team, including registered dietitians [91], could use simple surveys of chemosensation, mirroring the National Health and Nutrition Examination Survey [60], as well as a liking survey [41] to help track diet-related outcomes associated with weight loss after bariatric surgery.

## Figures and Tables

**Table 1:** Characteristics of females awaiting bariatric surgery (Pre-op) and those 1-year post-surgery (RYGB = Roux en Y Gastric Bypass; SG = Sleeve Gastrectomy), grouped as successful or unsuccessful at weight loss based on percent excess weight loss (%Excess Weight Loss (%EWL); cut off at 65%) or percent weight loss (%Weight Loss (%WL); cut off at 27%).

Variables	Pre-Op *n* = 49	Post-Surg *n* = 48	Success %EWL *n* = 23	Unsuccess %EWL *n* = 25	Success %WL *n* = 26	Unsuccess %WL *n* = 22
**Age ^†^**	45.7 ± 1.6	48.44 ± 1.6	47.8 ± 1.8	49.0 ± 2.5	46.4 ± 2.2	50.8 ± 2.2
**Race ^¥^**						
Black	25	21	13	28	15	27
Other	4	10	17	4	15	5
White	71	69	70	68	70	68
**Ethnicity ^¥^**						
Hispanic	16	13	22	4	19	5
Not-Hispanic	84	87	78	96	82	95
**Surgery Type ^¥^**						
RYGB	51	50	48	52	59	38
SG	49	50	52	48	41	62
**Pre-Op ^†^**						
Weight (lbs)	266.5 ± 6.7	273.4 ± 8.8	238.4 ± 6.4 ^b^	305.6 ± 12.7 ^a^	277.8 ± 14.9	268.1 ± 7.5
BMI ^††^	45.7 ± 1.2	47.0 ± 1.6	41.0 ± 1.3 ^b^	52.6 ± 2.4 ^a^	48.0 ± 2.8	45.9 ± 1.4
**Post-Surgery ^†^ (1 year)**						
Weight (lbs)		198.3 ± 7.9	156.1 ± 4.3 ^b^	240.6 ± 8.0 ^a^	183.7 ± 11.7 ^b^	219.5 ± 6.8 ^a^
BMI ^††^		33.8 ± 1.4	26.4 ± 0.7 ^b^	41.2 ± 1.7 ^a^	31.4 ± 2.1 ^b^	37.3 ± 1.4 ^a^

Note: ^†^ mean ± standard error of the mean; ^¥^ percentage of group; ^††^ Body Mass Index (kg/m2); ^a^ versus; ^b^ are significantly different; *p* < 0.01.

**Table 2:** Joint distribution of females who are post-bariatric surgery classified as successful or unsuccessful at weight loss at 1-year by percent weight loss (%WL; cut off at 27%) or percent excess weight loss (%EWL; cut off at 65%).

%WL				
		Successful	Unsuccessful	Total
**%EWL**	Successful	19	4	23
	Unsuccessful	7	18	25
	Total	26	22	48

**Table 3:** Self-reported smell function in females awaiting bariatric surgery (Pre-Op; *n* = 49) and Post-Op (*n* = 48) with 1-year weight loss characterized as successful or unsuccessful based on percent excess weight loss (%EWL) or percent weight loss (%WL) with comparison to data from the National Health and Nutrition Examination Survey (NHANES).

Smell ^†^	Post-Op - %EWL		Post-Op - %WL		Pre-Op	NHANES
	Successful	Unsuccessful	Successful	Unsuccessful		
Problem since surgery						
Yes	9	8	10	5		
No	87	92	85	95		
Don’t Know	4	0	5	0		
Problem in past year						
Yes					9	8
No					91	92
Description since surgery						
Excellent	35	42	41	35		
Good	35	46	37	45		
Little trouble	17	8	11	15		
Moderate trouble	4	0	4	0		
A lot of trouble	0	0	0	0		
Loss smell	0	0	0	0		
Don’t Know	9	4	7	5		
Change since 25 years						
Better					22	6
Worse					7	14
No Change					65	79
Don’t Know					7	0
Change since surgery						
Better	22	24	30	14		
Worse	4	4	4	5		
No Change	74	68	67	76		
Don’t Know	0	4	0	5		
Specific problem since surgery						
None	65	68	59	76		
less able	0	8	0	9		
parosmia or phantom	26	8	33	5		
smell stronger/make sick or anxious	4	12	4	5		
Don’t Know	4	4	4	5		
Specific problem in past year						
None					72	
less able					4	
parosmia or phantom					11	7
smell stronger/make sick or anxious					13	
Don’t Know					0	

^†^ Grey shaded areas are significantly different by chi square or Fisher’s exact testing.

**Table 4:** Self-reported taste function in females awaiting bariatric surgery (Pre-Op; *n* = 49) and Post-Op (*n* = 48) with 1-year weight loss characterized as successful or unsuccessful based on percent excess weight loss (%EWL) or percent weight loss (%WL) with comparison to data from the National Health and Nutrition Examination Survey (NHANES).

Taste ^†^	Post-Op - EWL		Post-Op - % Wt Loss		Pre-Op	NHANES
	Successful	Unsuccessful	Successful	Unsuccessful		
Problem since surgery						
Yes	26	28	30	24		
No	70	68	66	71		
Don’t Know	4	4	4	5		
Problem in past year						
Yes					6	5
No					94	95
Description since surgery						
Excellent	43	42	48	35		
Good	39	33	33	40		
Little trouble	9	17	11	15		
Moderate trouble	4	4	4	5		
A lot of trouble	4	4	4	5		
Loss smell	0	0	0	0		
Don’t Know	0	0	0	0		
Change since 25 yrs across each taste quality						
Better	21.7 to 39.1	8.7 to 30.4	42.3	23.8	17–26	4–8
Worse	8.7 to 21.7	13.0 to 17.4	15.4	19.1	9–13	7–13
No Change	52.2 to 60.9	60.1 to 82.6	42.3	57.1	60–64	87–92
Don’t Know	0 to 8.7	0 to 17.4	0.0	0.0	6–9	<1
Ability to taste food flavor as good as when younger						
Yes					85	92
No					6	7
Don’t Know					8	1
Change since surgery across each taste quality						
Better	22–44	8–28	26–44	0–24		
Worse	9–22	12–20	11–19	14–19		
No Change	32–61	56–72	41–59	57–71		
Don’t Know	0–9	0–12	0–7	0–14		
Ability to taste food flavor as good since surgery						
Yes	82	88	89	81		
No	9	8	4	14		
Don’t Know	9	4	7	5		
Specific problem since surgery						
None	39	52	37	55		
Can’t taste some things	0	8	4	5		
Things don’t taste right	48	20	45	27		
taste stronger	13	20	15	14		
Specific problem in past year						
None					87	
less able					0	
dysgeusia					7	6
stronger					2	
Don’t Know					4	

^†^ Grey shaded areas are significantly different by chi square or Fisher’s exact testing.

**Table 5:** The percentage of 6-n-propylthiouracil (PROP) taster groups between women with morbid obesity considering bariatric surgery (Pre-Op) and a separate group of women at 1-year post-bariatric surgery (Post-surg), and those post-surgery who were successful versus unsuccessful at weight loss, defined by percent excess weight loss (%EWL) or percent weight loss (%WL).

			%EWL		%WL	
	Pre-Op	Post-Surg	Successful	Unsuccessful	Successful	Unsuccessful
Nontaster	29	26	30	21	27	24
Medium taster	51	45	43	46	46	43
Supertaster	20	30	26	33	27	33

**Table 6:** Conceptual groups generated from a food liking survey and internal consistency scores tested with Cronbach’s Alpha in women who were pre- and 1-year post bariatric surgery.

Group	Cronbach’s α	Mean ± SEM
**Physical Activity—**bicycling, working up a sweat, playing sports, exercising with others, exercising alone, going to the gym, taking the stairs	0.84	15.12 ± 3.8
**Sweet foods—**cookies, cake, or pie, jam or jelly, ice cream, icing	0.81	25.46 ± 4.2
**Alcohol—**vodka, gin, or scotch, white wine, red wine, beer	0.79	−31.54 ± 5.2
**High-Fat Protein—**bacon, pizza, fried chicken, sausage, fried fish, pork chops, charred meat, cheddar cheese	0.77	34.52 ± 3.1
**Sour—**sour pickles, lemon, vinegar	0.72	13.0 ± 4.3
**Vegetable—**eggplant, spinach or greens, beets, sautéed mushrooms, asparagus, raw carrots, broccoli, tomatoes	0.71	28.7 ± 3.3
**Refined Carbohydrate—**crackers, white potato, cornflakes, white rice, pasta, bagel or rolls	0.70	25.2 ± 3.1
**Fruit—**strawberries, pineapple, cherries, pear, melon, banana	0.70	48.2 ± 3.0
**Spicy/flavorful —**Tabasco sauce, raw onion, chili pepper, garlic, soy sauce, blue cheese, dark chocolate	0.67	−2.8 ± 3.4
**Fat—**olive oil, salad dressing, mayonnaise, butter	0.66	28.9 ± 3.3
**Salt—**salting foods, ham, pretzels, olives, tortilla or potato chips, French fries	0.65	20.2 ± 3.3
**Sweet Drinks—**orange juice, coffee drinks, sugar-sweetened coffee or tea, soda	0.58	1.43 ± 4.3
**Bitter—**tea, grapefruit juice, black coffee, unsweetened iced tea	0.56	11.2 ± 3.6
**Pleasant—**hearing favorite music, going to a coffee shop, going to a pub or bar, smell of cut grass, cooling off on a hot day, television	0.55	43.2 ± 2.7
**Low-Fat Protein—**tuna or salmon, baked chicken, plain yogurt, shrimp	0.52	38.1 ± 3.4
**Fiber—**fiber bar, oatmeal, lentils or beans, whole wheat bread	0.40	28.9 ± 3.0
**Unpleasant—**glare of headlights, car accident, seeing a mouse at home	0.37	−72.0 ± 2.3
